# Study on the T-Cell Immune Response in Individuals With HIV and Toxoplasmosis Using ELISPOT

**DOI:** 10.1155/ipid/9514227

**Published:** 2025-07-17

**Authors:** Iskra Georgieva Rainova, Rumen Nenkov Harizanov, Yana Dimitrova Todorova, Mihaela Vanyova Videnova, Eleonora Marinova Kaneva, Raina Borisova Enikova, Nina Dimitrova Tsvetkova

**Affiliations:** ^1^Department of Parasitology and Tropical Medicine, National Centre of Infectious and Parasitic Diseases (NCIPD), Sofia, Bulgaria; ^2^Department of Immunology, National Centre of Infectious and Parasitic Diseases, Sofia, Bulgaria

**Keywords:** CD4/CD8 ratio, diagnostics, ELISA, ELISPOT, HIV, immune response, specific antibodies, toxoplasmosis

## Abstract

**Introduction:** The intracellular parasite *Toxoplasma gondii* stimulates the human immune system, resulting in the activation of both cellular and humoral immune responses. In HIV-infected individuals, latent *Toxoplasma* infection can reactivate, resulting in toxoplasmosis encephalitis (TE). Detection of specific memory T cells in such patients will prevent the risk of toxoplasmosis-related complications. ELISPOT assesses CD4+ and CD8+ T cell responses to antigens, and facilitates the identification of *T. gondii*-specific IFN-γ producing memory T cells in patients with both toxoplasmosis and HIV.

**Patients and Methods:** ELISA was used to test 104 blood samples from HIV + individuals for *Toxoplasma* antibodies. Peripheral blood mononuclear cells were isolated from the blood samples of the toxoplasmosis-positive HIV-infected patients and used to analyze the T-cell immune response. Peptides from the *T. gondii* were selected to stimulate CD4+ and CD8+ T cells when performing the ELISPOT.

**Results:** Serological data for toxoplasmosis was identified in 29 (27.6%) of the total number of patients. A significant difference was observed in the CD4+ T cell count between HIV-positive patients with and without toxoplasmosis. Seven of the HIV-infected patients with toxoplasmosis had a low CD4+/CD8+ T cell ratio. After performing a 16–20 h ELISPOT with peptide stimulation to investigate the presence of specific IFN-γ-producing cells in these seven patients, no IFN-γ-secreting cells were detected. Subsequently, a modified method was used, in which the immune cells were stimulated for a period of 5 days. At the end of this stimulation, all samples from HIV-infected patients with toxoplasmosis were ELISPOT positive, with a mean of 32 and 45 spots per well, respectively.

**Conclusion:** It is important to monitor patients with HIV, toxoplasmosis, and immunodeficiency. This can help prevent complications such as TE. A modified ELISPOT protocol may be required to determine the specific cell-mediated response in immunocompromised patients.

## 1. Introduction

Toxoplasmosis is an infection caused by the single-celled coccidian parasite, *Toxoplasma gondii*. This protozoan has a wide range of hosts, infecting 30 species of birds and 300 species of mammals, including humans [1]. However, its definitive hosts are members of the Felidae family (cats).

The disease is classified into two main categories: congenital (passed from mother to child) and acquired (contracted after birth). Depending on whether the infection is newly acquired or has been present for some time, it can also be categorized as acute or chronic (latent).

In individuals with a healthy immune system, toxoplasmosis typically presents without symptoms or as lymphadenitis (swelling of the lymph nodes). However, those infected with the human immunodeficiency virus (HIV) are at a higher risk of developing severe diseases, such as toxoplasmic encephalitis (TE). The relationship between HIV-positive patients and toxoplasmosis as an opportunistic infection has been extensively investigated. Since the advent of the 21st century, 500 HIV-positive patients have been screened for toxoplasmosis at the NCIPD, with 168 (34%) testing positive for specific IgG antibodies. Despite the administration of antiretroviral therapy (ART) and prophylaxis for toxoplasmosis with trimethoprim/sulfamethoxazole, 12 cases (2.4%) of clinically evident cerebral toxoplasmosis, due to the reactivation of latent infection, were diagnosed and reported during this period [2–5]. In a separate study, Kodym et al. identified nine cases of toxoplasma encephalitis in individuals infected with the HIV over a period of 15 years [6]. In Brazil, Xavier et al. demonstrated that with a seroprevalence of 80%, toxoplasma encephalitis was observed in 5.6% of HIV patients [7].

Infection with *T*. *gondii* triggers a strong and sustained immune response that includes both cellular and humoral components. The cellular response involves the production of pro-inflammatory cytokines such as interleukin-12 (IL-12), interferon-gamma (IFN-γ), and tumor necrosis factor (TNF). These cytokines are crucial for eliminating the parasite from infected cells. This robust cell-mediated immune response also leads to the production of high levels of specific antibodies in the bloodstream. The presence of specific IgG, IgM, and IgA antibodies can be used to identify individuals who have been exposed to the parasite and to distinguish between recent and latent infections. In HIV infected patients, peripheral blood mononuclear cells (PBMCs) show decreased levels of IL-12 and IFN-γ in response to this parasite. Enzyme-linked immunospot (ELISPOT) is a technique for quantifying the number of T cells specific to antigens, thereby elucidating host-pathogen relationships [8]. ELISPOT is also a valuable tool for investigating IFN-γ production and determining the T-cell immune response to *T. gondii* in blood samples from HIV patients [9].

The present study aims to detect specific antibodies to *Toxoplasma gondii* in HIV-infected patients and evaluate their cell-mediated immune response using an IFN-γ-based ELISPOT assay.

## 2. Patients and Methods

### 2.1. Patients

The study included 104 patients diagnosed with HIV infection, aged between 14 and 62 years (mean age 40 years), comprising 91 males and 13 females. All of them were required to provide a peripheral blood sample for routine immunological monitoring at the National Center of Infectious and Parasitic Diseases (NCIPD), Sofia during the 2022-2023 period. Following the provision of informed consent, the samples were used for the purposes of the study. The immunological monitoring included the determination of the absolute count (AC) of CD4+ and CD8+ T cells. This was performed by direct flow cytometry (Multitest, BD Trucount, FACS Canto II, USA). Data on ART were available for 92 patients, and advanced immunodeficiency (CD4 < 200 cells/μL) was found in 10 of the patients.

There was a paucity of data regarding the toxoplasmosis status of all patients prior to the commencement of the tests.

### 2.2. Ethical Considerations

The study was reviewed and approved by the Institutional Review Board (IRB) 00006384 and informed consent was obtained from the patients. No information that could identify the patients in the study was used.

### 2.3. Methods

#### 2.3.1. Determination of Specific Antitoxoplasma Antibodies

The presence of *Toxoplasma* IgG, IgM, and IgA antibodies was determined by ELISA (Test Line Clinical Diagnostics, Czech Republic). The concentration of specific antibodies was calculated using the index of positivity (IP), which is the ratio between the absorbance of the test sample and the cut-off calibrator included in the kit. The result was read using an ELISA reader at a wavelength of 450 nm. Serum samples were run in duplicate, with the final result calculated as the average between the two readings of each sample. According to the manufacturer's instructions, results are negative if the index values are less than 0.9, borderline if they are between 0.9 and 1.1, and positive if the values exceed 1.1.

#### 2.3.2. Isolation of PBMCs

To study the T-cell immune response in HIV patients with serological data indicating the presence of *Toxoplasma* infection, PBMCs were isolated by density gradient centrifugation using Ficoll (1.077 g/mL, Capricon Scientific, Germany). After the isolation process, the cells were either tested or cryopreserved in a solution containing fetal bovine serum (FBS; Corning) and dimethyl sulfoxide (DMSO; Sigma Aldrich, USA) at a concentration of 10% until needed.

#### 2.3.3. Peptides

Unlike B lymphocytes, T cells are able to recognise antigen after they have been processed by antigen-presenting cells into peptide fragments of 8–25 amino acids in length. Class II Major Tissue Compatibility complex molecules for CD4+ lymphocytes and class I facilitate this process for CD8+ T cells. The selection of peptide antigens suitable for stimulation is crucial for the assessment of cell-mediated immune responses in individuals infected with *T. gondii*. After reviewing the literature, AS15 (AVEIKRPVPGTAPPS) was chosen to stimulate CD4+ T cells, while GRA6 (CLHPERVNVFDY) was selected for the stimulation of CD8+ lymphocytes [10–12]. The peptides were synthesised with > 98% purity using synthetic biomolecules from Thermo Fisher Scientific (USA) and then lyophilised. The working concentration for both peptides was determined after a series of experiments at 10 μg/mL [13].

#### 2.3.4. Determination of Specific T Cells to *T. gondii*

A modified ELISPOT assay was employed for the specific detection of *T. gondii*-specific T cells [14, 15]. Briefly, isolated PBMCs at a dilution of 2.5 × 10^5^ from patients with serological evidence of toxoplasmosis and reported marked immune dysfunction were loaded in the wells of a flat-bottomed plate (Oxford Immunotec) coated with monoclonal antibody against IFN-γ. For each patient, ELISPOT was performed in duplicate. After loading, the cells were stimulated overnight at 37°C with 5% CO_2_ using the peptides AS15 and GRA6. The T cell response was measured by assessing the number of IFN-γ spot-forming cells (SFCs) per well. In a limited number of samples, this activation was preceded by a 5-day stimulation with the same *T. gondii* peptides and IL-2, which is a cytokine known as a T-cell growth factor and also increases the sensitivity of ELISPOT [16, 17].

The results were evaluated using a digital microscope (Veho VMS-001, UK) and an ELISPOT reader (AID classic, Germany).

To ensure accuracy, unstimulated cells were used as the negative control, while cells stimulated with phytohaemagglutinin (PHA) served as the positive control.

### 2.4. Statistical Analysis

**Figure 1:**
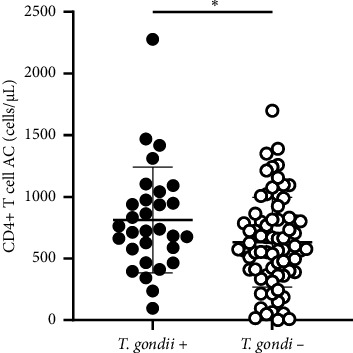
Comparison between the number of CD4+ cells in patients with and without *T. gondii* infection (^∗^*p* value less than 0.05 but greater than 0.01).

The Fisher's exact test was utilized for the data analysis, with *p* values lower than 0.05 were considered as statistically significant. To ascertain whether a statistically significant difference exists in the number of CD4 T cells between patients with evidence of toxoplasma infection and uninfected patients, the Mann–Whitney test was used. Correlations are expressed as Spearman's rank correlation values.  Figure 1 and statistical tests were performed using GraphPad Prism (v8.0.2).

## 3. Results

A total of 104 patients diagnosed with HIV were tested serologically for the presence of *Toxoplasma* antibodies. Specific IgG antibodies were detected in 29 patients (27.6%), which included 28 males and one female. Furthermore, specific IgM antibodies, indicative of an acute infection, were found in three patients. To determine the time of infection in those with positive IgM results, tests for specific IgA antibodies were conducted, yielding negative results. Moreover, an assessment of IgG avidity in these patients indicated a high level, suggesting that the *Toxoplasma* infection was acquired approximately 4–6 months prior. The ELISA testing results indicated that out of 28 seropositive patients, 26 (89.7%) had serological profiles consistent with latent infection, while three (10.3%) had recently acquired toxoplasmosis.

The ratio between sample extinction and the cut-off (IP) was observed to range from 1.1 to 1.9 in eight tested samples, with a mean value of 1.45 ± 0.25 SD. Among these, seven samples from HIV-infected individuals exhibited specific IgG antibody values ranging from 2.0 to 2.9, with a mean value of 2.37 ± 0.26 SD. Additionally, in 15 samples, specific IgG antibody values exceeded 3.0, with a mean value of 3.64 ± 0.65 SD. The patients who tested positive for *Toxoplasma* IgM antibodies were included in this latter group.

A comparison of the mean (±SD) values of CD4+ T cell counts of HIV patients positive for *T. gondii* (*n* = 29) (with IgG and IgM specific antibodies) (CD4+ 812 ± 430 cells/μL) and HIV patients negative for *Toxoplasma* infection (*n* = 74) (CD4+ 602 ± 370 cells/μL) revealed a significant difference (*p* < 0.05) (Figure 1).

Due to the potential for reactivation of *Toxoplasma* infection in patients with HIV, this study aimed to evaluate the risk of complications in individuals with a low CD4+ T cell count and a CD4+/CD8+ T cell ratio. A low CD4+/CD8+ ratio (≤ 0.3–0.4) indicates a higher risk of reactivation and complications for HIV patients with toxoplasmosis. This ratio acts as a prognostic marker for individuals infected with HIV, with lower values linked to worse outcomes. [18].

The results of the study indicated that there was a risk of reactivation of latent toxoplasmosis in one patient who was not on ART, had a high specific IgG antibody level (IP 3.0), and had an absolute CD4+ T lymphocyte count of 96 cells/μL.

Seven HIV-infected patients who tested positive for toxoplasmosis showed low CD4+/CD8+ T cell ratios during immunological monitoring. Among these patients, two had ratios below 0.1, two had ratios of 0.2, and three had a ratio of 0.3. This group was analyzed to determine the presence of specific IFN-γ-producing cells. Notably, one patient who had not received ART was included in this analysis.

**Figure 2:**
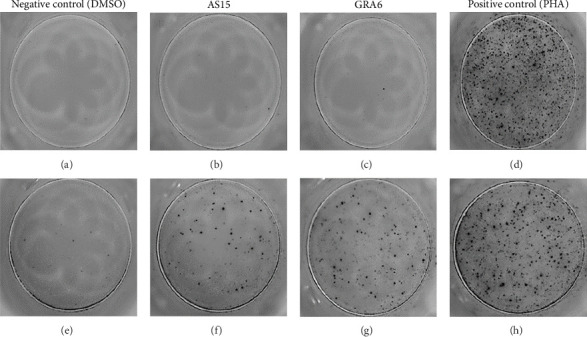
Results of the ELISPOT analysis, conducted after 16–20 h, and five days of stimulation with specific peptides derived from *T. gondii*. (a), (b), (c), (d)—ELISPOT results during the first step for 16–20 h. (a) Negative control (unstimulated cell with DMSO); (b), (c) stimulated with peptides AS15 and GRA6 cells; (d) positive control (cell stimulated with phytohaemagglutinin (PHA). (e), (f), (g), (h)—ELISPOT results after 5 days stimulation in the presence of IL-2. (e) Negative control (unstimulated cell); (f), (g) stimulated with peptides AS15, GRA6, and IL-2 cells; (h) positive control (PHA).

The analysis of T-cell reactivity using ELISPOT revealed the absence of IFN-γ-secreting cells following 16–20 h of stimulation with AS15 and GRA6 peptides. In accordance with the protocol modification proposed by Coughlan et al. (2015), PBMCs were stimulated also for 5 days with AS15, GRA6, and IL-2 (IL-2). Following a five-day incubation period, the cells were transferred to an ELISPOT plate and re-stimulated with the specific peptides for 16–20 h at 37°C in a CO_2_ incubator. The results demonstrated that all tested samples positive for toxoplasmosis were positive in ELISPOT, with a mean of 32 and 45 SFCs/10^6^ PBMC per well for AS15- and GRA6-specific T cells, respectively (Figure 2). We counted the spots under a digital microscope. However, when there were more than 20 spots per well, we also used an ELISPOT reader for a more accurate count.

Using Spearman's correlation test, we examined the relationship between the titer of antibodies against *Toxoplasma gondii*, the CD4/CD8 ratio, and the ELISPOT data. The results revealed a strong connection between the levels of specific *Toxoplasma* antibodies measured by ELISA and ELISPOT, conducted with both peptides AS15 and GRA6 (*r* = 0.99 for both peptides). The analysis of the relationship between the ELISPOT results conducted after stimulation with AS15 and GRA6 and the ratio of CD4+/CD8+ cells showed a moderately strong correlation between these variables (*r* = 0.66 for both peptides).

## 4. Discussion

To investigate *Toxoplasma* infection, we collected blood samples from 104 HIV-positive individuals who were undergoing routine immunological monitoring of their cellular immune status. The samples were tested serologically using ELISA to detect specific antibodies against *T. gondii*. The results indicated that 29 individuals (27.6%) exhibited evidence of *Toxoplasma* infection. Among these 26 individuals (89.7%) demonstrated a latent form, as indicated by specific IgG antibodies. Additionally, three individuals (10.3%) showed the presence of both *Toxoplasma* IgG and IgM antibodies, suggesting a recent infection. The serological results indicated that 15 patients (51.7% of those with seropositivity) had *Toxoplasma* IgG antibody levels above the cut-off value of 3.0, which is indicative of a potential risk for the reactivation of the *Toxoplasma i*nfection and a more severe clinical course.

Conversely, the immunological monitoring, which included the determination of the absolute number of CD4 and CD8 T cells, as well as their ratio, demonstrated that seven (24%) of HIV-infected individuals with antibodies to *T. gondii* also exhibited a risk of developing a severe *Toxoplasma* infection. The specific antibody values were ≥ 3.0 in three patients, while in the other four they were between 2.0 and 2.9. These findings are consistent with the hypothesis put forth by other researchers that a high level of IgG antibodies may serve as a predictive factor for the development of TE [19].

We conducted a study on T-cell function by measuring the cytokine production of IFN-gamma in patients with HIV and toxoplasmosis, particularly those showing signs of immune suppression (CD4/CD8 < 0.4). This was done using a modified ELISPOT method along with specific antigen stimulation. The initial results indicated that stimulation with the peptides AS15 and GRA6 for 16–20 h did not generate an immune response. This lack of response is likely due to a deficiency of effector T cells in the patient's peripheral blood. However, after a 5-day culture period with specific stimulation and the addition of IL-2, followed by re-stimulation for another 16–20 h, we observed a significant number of IFN-γ spots. According to Dubey et al. (2007), a positive response is having ≥ 55 spots per million cells, or at least four times the amount seen in the negative control [20]. Since there were no background spots in the negative control, we consider the results valid, especially given the context of the two concurrent infections (HIV and toxoplasmosis) and the associated immune suppression. The inclusion of the AS15 peptide in the ELISPOT assay was due to its designation as the first peptide shown to stimulate CD4 T cells. That makes it a valuable tool for investigating CD4 T cell responses in patients with toxoplasmosis. [11].

The literature indicates that GRA6-I/III with part VFDY is a highly immunogenic epitope [21]. However, there is a paucity of information on the ELISPOT test used to assess the naturally acquired immune response against *T. gondii* antigens [8]. The use of ELISPOT in toxoplasmosis is primarily limited to studies aimed at cell responses to candidate vaccine epitopes, with only a few available for diagnosis [22–24]. The results obtained by our research team demonstrate that the ELISPOT test with the specified peptides detects *Toxoplasma gondii*-specific IFN-γ-producing memory T cells in the peripheral blood of HIV-positive individuals. The presence of memory T cells in patients with HIV, toxoplasmosis, and evidence of significant immunodeficiency may serve to assess the possibility of recurrence of this opportunistic infection and/or the development of severe complications such as TE. This assumption is supported by strong correlation between *Toxoplasma* antibody titers, ELISPOT data after stimulation with the peptides AS15 and GRA6, and the CD4+/CD8+ cell ratio. In a 2007 study, Hoffman et al. evaluated the effect of highly active ART (HAART) and *T. gondii*-specific immune reactions on the occurrence of TE [9]. During the study, patients with an acute TE episode or relapse had a significantly lower incidence of *Toxoplasma*-specific activated T cells than patients who discontinued maintenance therapy and were without relapse. The authors suggest that assessing specific immune responses to this parasite using ELISPOT may determine the individual risk of developing TE.

## 5. Conclusions

The findings of our preliminary investigation suggest that it is imperative to implement serological screening for toxoplasmosis, particularly among newly diagnosed HIV-positive individuals. It is also crucial to monitor the dynamics of specific antibodies in registered patients and exhibit evidence of immune breakdown. That will facilitate the prophylaxis of infectious diseases and prevent the reactivation of latent infections or the development of complications of existing infections. Furthermore, a modified ELISPOT protocol may be employed to ascertain the presence of a cell-mediated immune response to *T. gondii* in immunosuppressed patients. In individuals with compromised immunity, ELISPOT is a more valuable tool than other immunological methods, as it allows for the monitoring of antigen-specific T cells and the measurement of a low number of T-lymphocytes [25]. To ascertain the efficacy of the technique in evaluating the likelihood of potential occurrence of cerebral toxoplasmosis, we have formulated a longitudinal study model, incorporating a cohort study of groups of HIV-infected patients.

## Data Availability

The data that support the findings of this study are available from the corresponding author upon reasonable request.
